# Retraction Note: Clinico-histopathologic and outcome features of cutaneous infundibular keratinizing acanthoma: a case report and literature review

**DOI:** 10.1186/s12957-016-1032-0

**Published:** 2016-11-04

**Authors:** Abbas Tavasoly, Hossein Gholami, Amir Rostami, Ali Anissian, Seyed Rashid Touni, Pooyan Khaleghian, Aram Mokarizadeh, Javad Javanbakht, Alireza Nasoori

**Affiliations:** 1 Department of Pathology, Faculty of Veterinary Medicine, Tehran University, Tehran, Iran; 2 Department of Clinical Science, Faculty of Veterinary Medicine, Tehran University, Tehran, Iran; 3 Department of Veterinary, College of Agriculture, Abhar Branch, Islamic Azad University, Abhar, Iran; 4 Faculty of Veterinary Medicine, Urmia University, Urmia, Iran; 5 Graduated of Faculty of Veterinary Medicine, Garmsar Branch, Islamic Azad University, Garmsar, Iran; 6 Department of Immunology, Faculty of Medicine, Kurdistan University of Medical Sciences, Sanandaj, Iran; 7 Graduate of Veterinary Medicine, Faculty of Veterinary Medicine, Karaj Branch, Islamic Azad University, Rajaee shahr, Moazen Boulevard, Karaj, Alborz Iran; 8 Department of Pathobiology, Faculty of Veterinary Medicine, Tehran University, Tehran, Iran

## Retraction

The Editor-in-Chief and Publisher have retracted this article [1] because the scientific integrity of the content cannot be guaranteed. An investigation by the Publisher found it to be one of a group of articles we have identified as showing evidence suggestive of attempts to subvert the peer review and publication system to inappropriately obtain or allocate authorship. This article showed evidence of plagiarism (most notably from the articles cited [2–5]) and authorship manipulation.
